# Direct-acting antiviral treatment significantly shaped the gut microbiota in chronic hepatitis C patients: a pilot study

**DOI:** 10.3389/fmicb.2025.1664447

**Published:** 2025-09-04

**Authors:** Nahla M. Elsherbiny, Omnia M. Kamal El-Din, Elham Ahmed Hassan, Helal F. Hetta, Roba Alatawy, Mostafa A. Sayed Ali, Fawaz E. Alanazi, Mohamed S. Abdel-Maksoud, Hashim M. Aljohani, Mohamed Saad Badary, Zienab Gaber Mahran, Marwa Abo Kresha, Khaled Abo Bakr, Hossam Abdelwahab, Mohammed Ramadan

**Affiliations:** ^1^Medical Microbiology and Immunology Department, Faculty of Medicine, Assiut University, Assiut, Egypt; ^2^Microbiology and Immunology Department, Faculty of Pharmacy, Assiut University, Assiut, Egypt; ^3^Gastroenterology and Tropical Medicine Department, Faculty of Medicine, Assiut University, Assiut, Egypt; ^4^Division of Microbiology, Immunology and Biotechnology, Department of Natural Products and Alternative Medicine, Faculty of Pharmacy, University of Tabuk, Tabuk, Saudi Arabia; ^5^Department of Medical Microbiology, Faculty of Medicine, University of Tabuk, Tabuk, Saudi Arabia; ^6^Molecular Microbiology and Infectious Diseases Research Unit, University of Tabuk, Tabuk, Saudi Arabia; ^7^Department of Pharmacy Practice, Faculty of Pharmacy, University of Tabuk, Tabuk, Saudi Arabia; ^8^Department of Pharmacology and Toxicology, Faculty of Pharmacy, University of Tabuk, Tabuk, Saudi Arabia; ^9^Department of Clinical Laboratory Sciences, College of Applied Medical Sciences, Taibah University, Madina, Saudi Arabia; ^10^Department of Pathology and Laboratory Medicine, College of Medicine, University of Cincinnati, Cincinnati, OH, United States; ^11^Internal Medicine Department, Faculty of Medicine, Assiut University, Assiut, Egypt; ^12^Microbiology and Immunology Department, Faculty of Pharmacy, Al-Azhar University, Assiut, Egypt

**Keywords:** chronic hepatitis C, direct-acting antivirals, gut microbiota, sustained virological response, relapse

## Abstract

**Background:**

Chronic hepatitis C (CHC) can be effectively cured with direct-acting antivirals (DAAs), yet the impact of CHC on the gut microbiota remains controversial, with limited research on changes in patients who achieve a sustained virological response (SVR) versus those who relapse.

**Aim:**

To investigate the impact of CHC on the gut microbiota and compare changes between patients who achieved SVR post-DAA treatment and those who relapsed.

**Methods:**

In this case-control study, 60 stool samples were collected from CHC patients (20 untreated, 20 post-DAAs SVR, and 20 relapsed patients) and 20 healthy individuals. The V3–V4 regions of the 16S rRNA gene were sequenced using MiSeq to analyze bacterial diversity and composition.

**Results:**

Compared with healthy participants, CHC patients presented significantly altered bacterial diversity. The microbial diversity of the SVR patients was similar to that of the controls (*p* = 0.45), whereas the microbial diversity of the relapsed patients was lower. The gut microbiota clearly clustered on the basis of disease status. Firmicutes were predominant in treated patients, whereas Bacteroidetes and Proteobacteria were enriched in the relapsed group. Compared with the other groups, the SVR group presented lower Actinobacteria and higher Cyanobacteria levels. Genus-level analysis revealed significant disease-dependent biomarkers and intermicrobial coexistence. *Prevotella, Bifidobacterium*, and *Lactobacillus* were more prevalent in relapsed patients, whereas *Bacteroides*, *Agathobacter,* and *Parabacteroides* were more abundant in controls. *Elusimicrobium*, *Christensenellaceae R-7*, *Catenibacterium*, *Oceanobacillus*, and *Candidatus Melainabacteria* were significantly more abundant in the SVR group.

**Conclusion:**

DAAs have a significant impact on the gut microbiota in CHC patients, resulting in distinct microbial patterns, biomarkers, and interactions. Successful HCV eradication restores bacterial diversity and reestablishes microbial communities resembling those in healthy individuals.

## 1 Introduction

Hepatitis C virus (HCV) infection remains a major global health challenge, with approximately 1.5 million new cases per year and a worldwide prevalence of 58 million (WHO Global Hepatitis Report, 2022). In Egypt, a large-scale screening campaign led by the Ministry of Health identified 1.1 million individuals with HCV viremia; 92% of whom initiated treatment with DAAs (Waked et al., 2020), which has significantly improved cure rates up to 95%, even in complex cases (Elnadry et al., 2018; Martinello et al., 2017; Nawaz et al., 2023). DAAs have significantly improved cure rates to up to 95%, even in complex cases (Nawaz et al., 2023; Fathalla Khattab et al., 2021).

The gut-liver axis facilitates communication between the gut microbiota and the liver via the portal vein, systemic circulation, and biliary tract (Ladenheim et al., 1995). This connection allows the liver to handle potentially harmful substances from the gut while the liver secretes bile into the intestines, facilitating bidirectional communication (Guilliams et al., 2022). Most bile acids are reabsorbed in the terminal ileum and recycled to the liver, with some being converted into secondary bile acids by the colonic microbiota before being reabsorbed (Grüner and Mattner, 2021). This communication is crucial for gastrointestinal health and disease management. Studies indicate that liver diseases disrupt bile acid homeostasis, leading to proinflammatory bacterial overgrowth, alterations in the microbial community and disease progression (Mouzaki et al., 2016; Kakiyama et al., 2013; Qin et al., 2014).

HCV infection negatively affects gut health by reducing beneficial species that produce short-chain fatty acids essential for intestinal barrier integrity and immune regulation (Wong et al., 2006). This dysbiosis increases gut permeability, contributing to liver damage and potentially advancing HCV infection to cirrhosis and hepatocellular carcinoma (Aly et al., 2016; Frumento and Ţălu, 2024).

Research on the impact of HCV infection on the gut microbiota, particularly in developing countries, is still limited. The mechanisms through which microbial dysbiosis affects disease progression remain unclear (Aly et al., 2016; Bajaj et al., 2016; Zheng et al., 2020; Sultan et al., 2021). While achieving a SVR is linked to improved outcomes, the effect of HCV eradication on gut dysbiosis in chronic hepatitis C (CHC) patients is still debated because of challenges in controlling external factors affecting the microbiota in humans compared with those in animal models (Martinello et al., 2023). Furthermore, the role of DAAs in altering the microbiota, particularly among relapsed patients, has not been thoroughly investigated. Therefore, this study aimed to investigate the impact of CHC on the gut microbiota and compare microbiota changes in CHC patients who achieved SVR post-DAA treatment with those in patients who experienced relapse.

## 2 Materials and methods

### 2.1 Study design

This case-control study was conducted at Assiut University Hospital, Assiut, Egypt, between January 2023 and June 2024. The aim of the current study was to assess the effects of CHC and its treatment (DAAs) on the gut microbiota. The study was approved by the Research Ethical Committee of the Faculty of Medicine, Assiut University (IRB 200392), conducted following the Declaration of Helsinki, and was registered with Clinical Trials. gov. (NCT06829966). Informed written consent was obtained from all participants before enrollment.

The sample size was calculated based on previously published studies of hepatitis C-associated gut microbiota (power of 0.8 and *α* < 0.05) using variations in alpha diversity, beta diversity, and the abundance of ASVs (amplicon sequence variants) or operational taxonomic units (OTUs) (Abd Alla et al., 2018). The total required sample size was approximately 60.

### 2.2 Study population

This study was conducted on 60 non-cirrhotic CHC patients: 20 treatment-naive patients (Non-Treated group), 20 patients who achieved SVR after 12 weeks of treatment with DAAs (specifically, treated with sofosbuvir and daclatasvir; SVR group), and 20 patients who relapsed following the completion of the same treatment regimen (relapsed group). Additionally, 20 healthy control subjects who were negative for hepatitis B virus (HBV) and hepatitis C virus (HCV) markers and matched for age, sex, and socioeconomic status were included in the study (Fahmy and El Sherbini, 1983). All participants were recruited from the Hepatitis Outpatient Clinic, Al-Rajhi Liver Center, Assiut University Hospital, Egypt.

Eligible patients were 18 years or older and had at least a 6-month history of HCV infection. These patients were classified as noncirrhotic based on clinical findings and imaging, including transient elastography (TE, FibroScan, Echosens, Paris, France); all patients had mild hepatic fibrosis (<7 kPa) as assessed by TE (Bonder and Afdhal, 2014). SVR was defined as undetectable HCV-RNA in the serum 3 months after treatment (Yoshida et al., 2015). Patients receiving antibiotic treatment, probiotics, or any other medical treatment influencing the gut microbiota 1 month before the start of the study as well as patients with any other viral infection, such as HBV or HIV, were excluded.

All participants underwent detailed medical history, clinical examination, abdominal ultrasound, transient elastography, and laboratory investigations, including complete blood count (CBC), liver function tests and serum creatinine.

### 2.3 Specimen collection

Fresh stool samples were collected in the morning from all participants and were processed within 1 h after defecation. Additionally, 5 mL of venous blood under aseptic conditions was collected from each patient for the estimation of the different laboratory parameters.

#### 2.3.1 Quantitative assessment of the RNA viral load

HCV-RNA levels were detected using real-time polymerase chain reaction (RT-PCR) (Bioline International, UK), with a lower limit of detection of 15 IU/mL.

### 2.4 Microbiota profiling

#### 2.4.1 DNA extraction

Immediately after collection, genomic DNA was extracted from the stool samples using the Invitrogen PureLink Microbiome DNA Purification Kit (Thermo Fisher Scientific, Cat #A29790) according to the manufacturer’s instructions.

#### 2.4.2 PCR amplification and 16S rRNA amplicon sequencing

PCR was conducted to amplify hypervariable regions V3–V4 of the 16S rRNA gene in 25 μL reactions with 0.8 μL of each forward and reverse primer (10 μM, Metabion, Germany), 3 μL of template DNA, and 12.5 μL of 1 × Hot Master Mix (Genedirex PCR supermix). The following primers with Illumina adapters (underlined) were used:

Forward primer 5′TCGTCGGCAGCGTCAGATGTGTATAAGAGACAGCCTACGGGNGGCWGCAG3′

Reverse Primer 5′GTCTCGTGGGCTCGGAGATGTGTATAAGAGACAGGACTACHVGGGTATCTAAT C 3′

The thermal cycling conditions were as follows: initial denaturation at 95°C for 3 min; 30 cycles of denaturation at 95°C for 30 s, annealing at 55°C for 30 s and extension at 72°C for 30 s; and a final extension at 72°C for 10 min (Illumina, 2013). The amplified products were sent to IGA Technology Services (Udine, Italy) for sequencing using the Illumina MiSeq platform according to the manufacturer’s instructions.

### 2.5 Data analysis of 16S rRNA gene sequencing and statistical analysis

The 16S rRNA gene sequence data were analyzed using the Quantitative Insights into Microbial Ecology 2 platform (QIIME2). After deduplicating sequences, denoising, and removing chimeras, operational taxonomic units (OTUs) were identified. Secondary analysis was conducted using the linear discriminant analysis of effect size (LEfSe), Greengenes13_8, and Microbiome Analyst for statistical and meta-analyses of microbiome data (Dhariwal et al., 2017).

Alpha diversity was analyzed using the number of observed species and the Shannon diversity index. Beta diversity analysis was also determined by Principal Coordinate Analysis (PCoA) based on weighted and unweighted UniFrac distances. The statistical significance of shifts in bacterial diversity was determined using the nonparametric Wilcoxon rank-sum test and the Kruskal–Wallis rank-sum test. The resulting *p* values were adjusted using the false discovery rate method (FDR) (Benjamini and Hochberg, 1995). The significance of sample clustering was assessed by Permutational Multivariate Analysis of Variance (Adonis R, package Vegan) (Anderson, 2001).

To identify the genera responsible for driving the shifts in microbiomes, DESeq2 was used to analyze all the genera in the dataset (FDR-corrected *p* value < 0.05). Spearman correlation distance was applied to assess correlations between bacterial taxa at different taxonomic levels (*r* ≥ ± 0.6, *p* ≤ 0.05) for dominant taxa (mean relative abundance ≥1.37; R package, Hmsic). Additionally, the enterotyping method was used to categorize the microbiomes into distinct clusters on the basis of specific genera. An OTU was classified as a core taxon if it was present in at least 80% of all samples of the whole dataset or in at least 80% of the samples within a specific study group. Linear discriminant analysis (LDA) effective size (LEfSe) was performed to define the potential biomarkers in each group (LDA scores >2.0, *α* = 0.05).

## 3 Results

### 3.1 Characteristics of the study groups

Sixty noncirrhotic CHC patients (28 males and 32 females with a mean age of 37.7 ± 13.4 years) and 20 healthy controls (9 males and 11 females with a mean age of 35.8 ± 10.2 years) were enrolled in the study. The demographic and laboratory characteristics of the patients are shown in Table 1.

**Table 1:** Demographic and laboratory characteristics of the patients.

Variable	Total CHC patients (*n* = 60)	Non-treated group (*n* = 20)	SVR group (*n* = 20)	Relapsed group (*n* = 20)	p*
Age (years)	37.74 ± 13.35	38.4 ± 16.6	30.8 ± 7.2	44.01 ± 14.4	0.452
Sex (male/female)	28/32 (50/50)	10/10 (50/50)	8/12 (40/60)	10/10 (50/50)	0.347
WBCs (×10^9^/L)	5.76 ± 1.52	6.38 ± 0.9	5.48 ± 1.3	5.43 ± 2.1	0.188
Hemoglobin (g/dl)	13.59 ± 0.83	13.46 ± 1.6	13.60 ± 1.1	13.73 ± 2.1	0.964
Platelet (×10^9^/L)	221.33 ± 62.3	229.75 ± 43.6	254.50 ± 54.1	179.75 ± 89.2	0.226
Total Bilirubin (mg/dl)	0.57 (0.24)	0.4 (0.3)	0.57 (0.14)	0.7 (0.1)	0.407
AST (U/L)	45.5 (20)	39 (65)	48.5 (60)	42.5 (40)	0.377
ALT (U/L)	44.25 (37)	49 (34)	43.5 (42)	80 (45)	0.165
Albumin (g/dl)	4.23 (1.74)	3.6 ± 1.6	4.6 ± 2.2	4.5 ± 1.3	0.321
INR	1.12 ± 0.50	1.01 ± 0.06	1.15 ± 0.4	1.20 ± 0.3	0.323
Serum Creatinine (mg/dl)	0.59 (0.1)	0.55 (0.18)	0.59 (0.19)	0.65 (0.2)	0.689
HCV-RNA (IU/mL)	607 (3,222.75)	1,021 (5,766)	—	148.5 (193)	0.045

The values are presented as frequencies (%), means ± standard deviations or medians [interquartile ranges (IQRs)].

*p-value for comparisons between variables in all groups analysis of variance (ANOVA) or Kruskal-Wallis H test and chi-square test for continuous parametric or non-parametric and dichotomous variables, respectively.

ALT, alanine aminotransferase; AST, aspartate aminotransferase; HCV, hepatitis C virus; INR, international normalized ratio; WBC, white blood cell.

#### 3.1.1 Sequence preprocessing and quality filtering

After quality checking, denoising, dereplication, merging, and removing chimeric sequences in QIIME2, 2,122,799 bacterial 16S rRNA reads (76.75% of the total reads) were obtained from 2,734,640 raw sequences.

### 3.2 Bacterial diversity analysis

**Figure 1:**
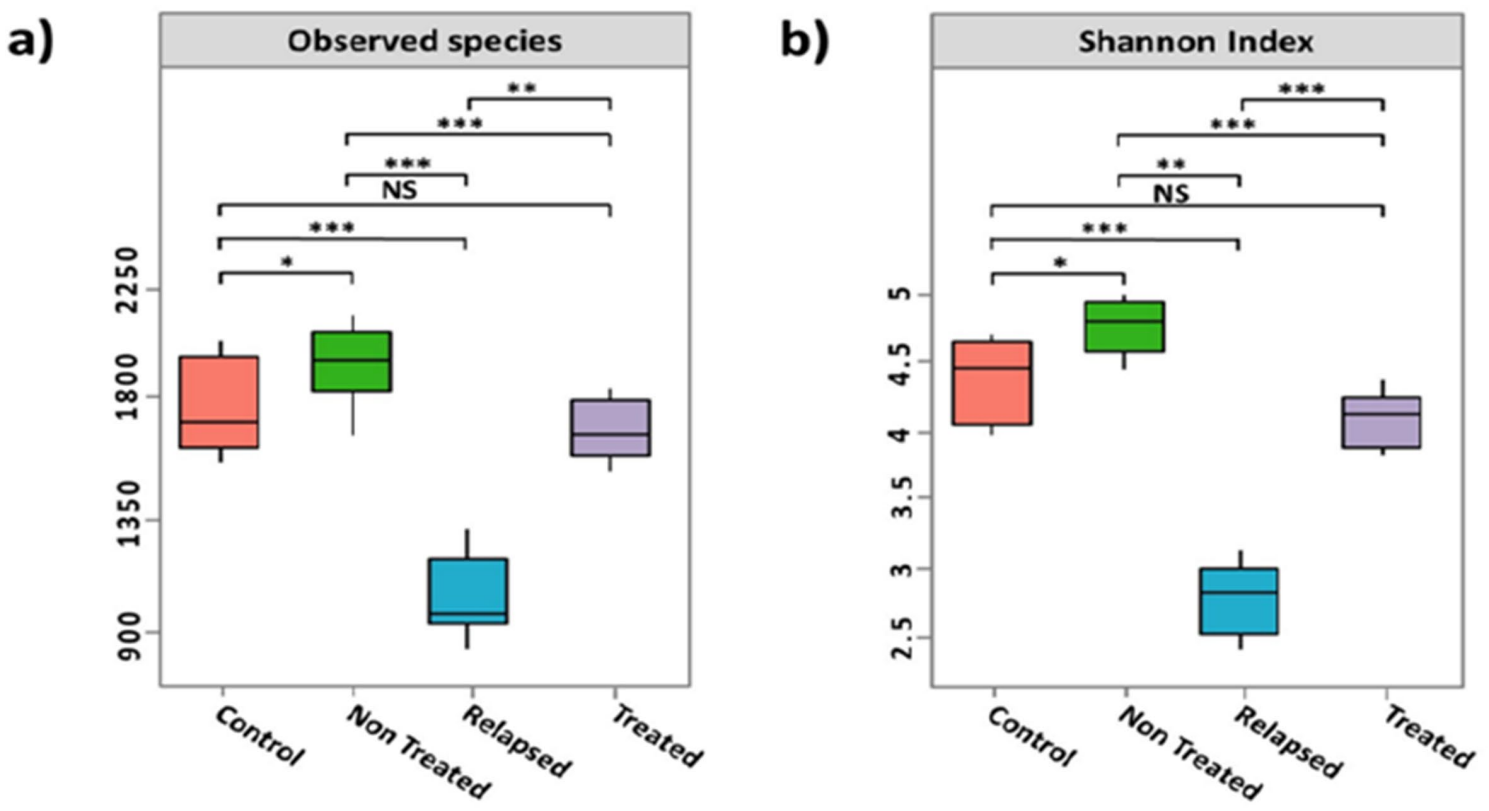
Alpha diversity indices of the gut microbiota among the studied groups. Each box plot displays the median, interquartile range (IQR), and range. The *x*-axis represents the study groups, and the *y*-axis represents the observed species in **(a)** and the Shannon index in **(b)**. Pairwise comparisons were conducted using the Wilcoxon rank-sum test. Significant differences are denoted by asterisks (*p < 0.05; **p < 0.01; ***p < 0.001). The box colors correspond to the study groups.

**Figure 2:**
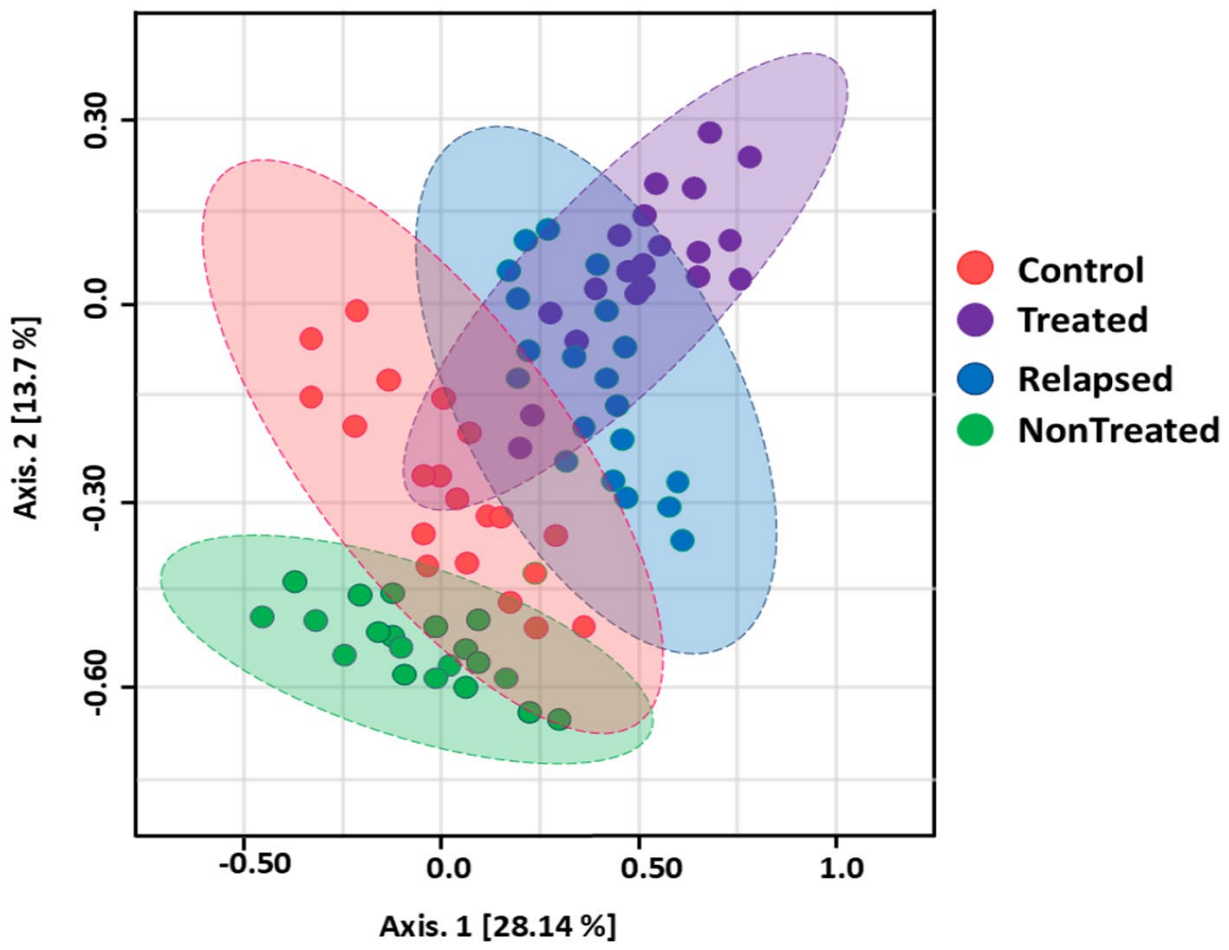
Principal coordinates analysis (PCoA) of the weighted UniFrac distance matrix of the gut microbial community structure. Each point on the PCoA plot represents a gut microbiota sample, with the *x*- and *y*-axes denoting the first and second coordinates, respectively. The percentages of community variation explained are shown in parentheses on each axis (28.14 and 13.7%, respectively). Ellipses indicate significant clustering (*p <* 0.001, PERMANOVA), with the color legend representing the study groups.

The taxonomic diversity of the gut microbiomes was assessed using various alpha diversity metrics, which estimate species richness by identifying 5,863 operational taxonomic units (OTUs). Compared with the other groups, the relapsed group presented significantly lower gut microbiome diversity. There was no significant difference in microbiome diversity between the SVR group and the control group (Figure 1). In addition, Principal Coordinate Analysis (PCoA) of weighted UniFrac distances revealed distinct clustering of the gut microbiomes of the four study groups (Figure 2).

### 3.3 Taxonomic profiles of the gut microbiome among the studied groups

**Figure 3:**
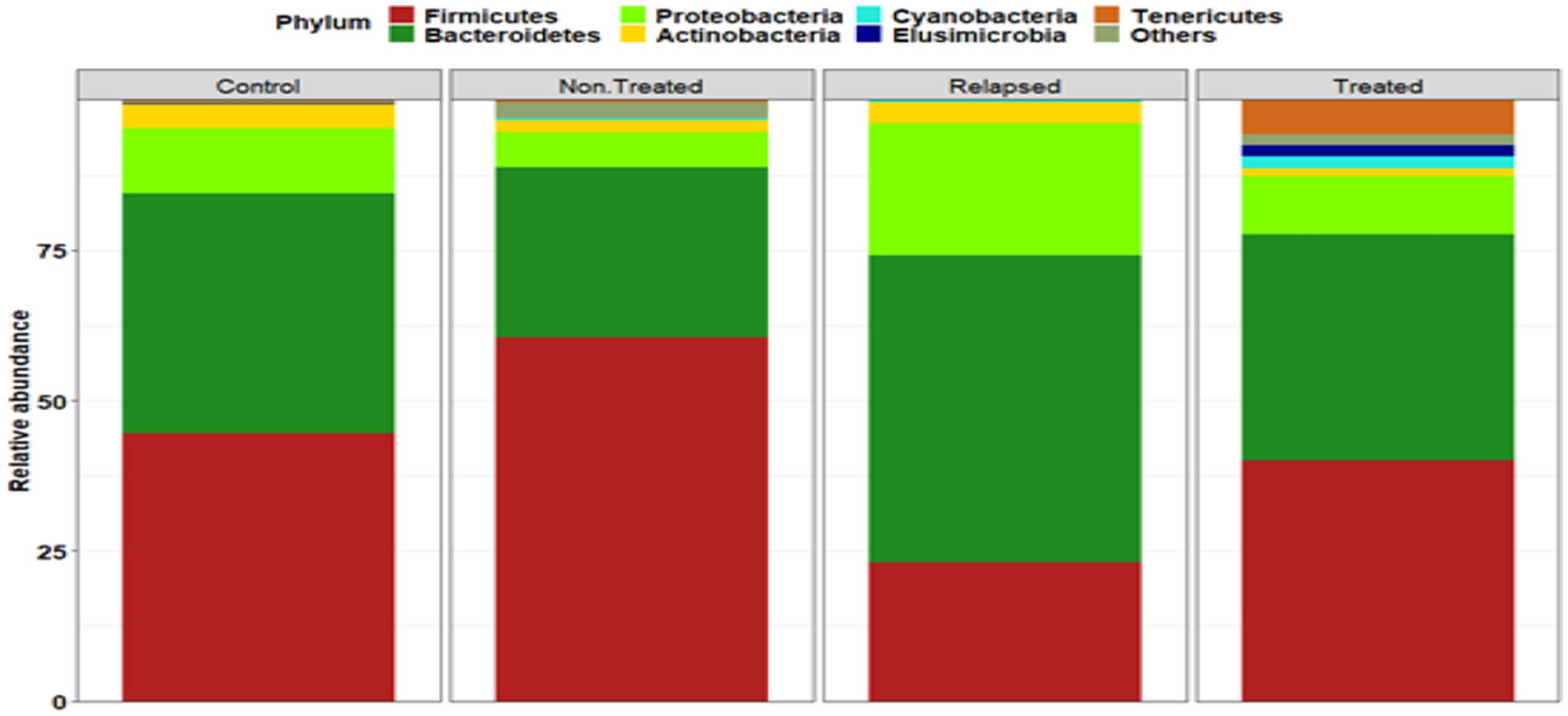
Phylum-level analysis of the gut microbiota of the studied groups. The stacked bar charts show the relative proportions of the predominant phyla in the gut microbiomes of various study groups. The *X*-axis indicates the relative abundance, whereas the *Y*-axis represents the study groups.

A total of 5,863 OTUs were identified in the gut microbiome, categorized into 26 phyla, 47 classes, 114 orders, 267 families, and 583 genera. The most prevalent phyla were Firmicutes and Bacteroidetes, with Proteobacteria, *Spirochaetes*, and *Cyanobacteria* also present (Figure 3). Firmicutes were most abundant in Non-Treated CHC patients, whereas Bacteroidetes and Proteobacteria were enriched in the relapsed group. Compared with those in the other groups, Actinobacteria levels were significantly lower in the SVR group, which also presented higher levels of *Cyanobacteria*. The Firmicutes/Bacteroidetes *(F/B)* ratios among the four groups were as follows: the control (1.57), Non-Treated CHC (2.27), treated (SVR) (1.11), and relapsed (0.49) groups. There were statistically significant differences in the F/B ratios among the groups, except between the control group and the treated group.

**Figure 4:**
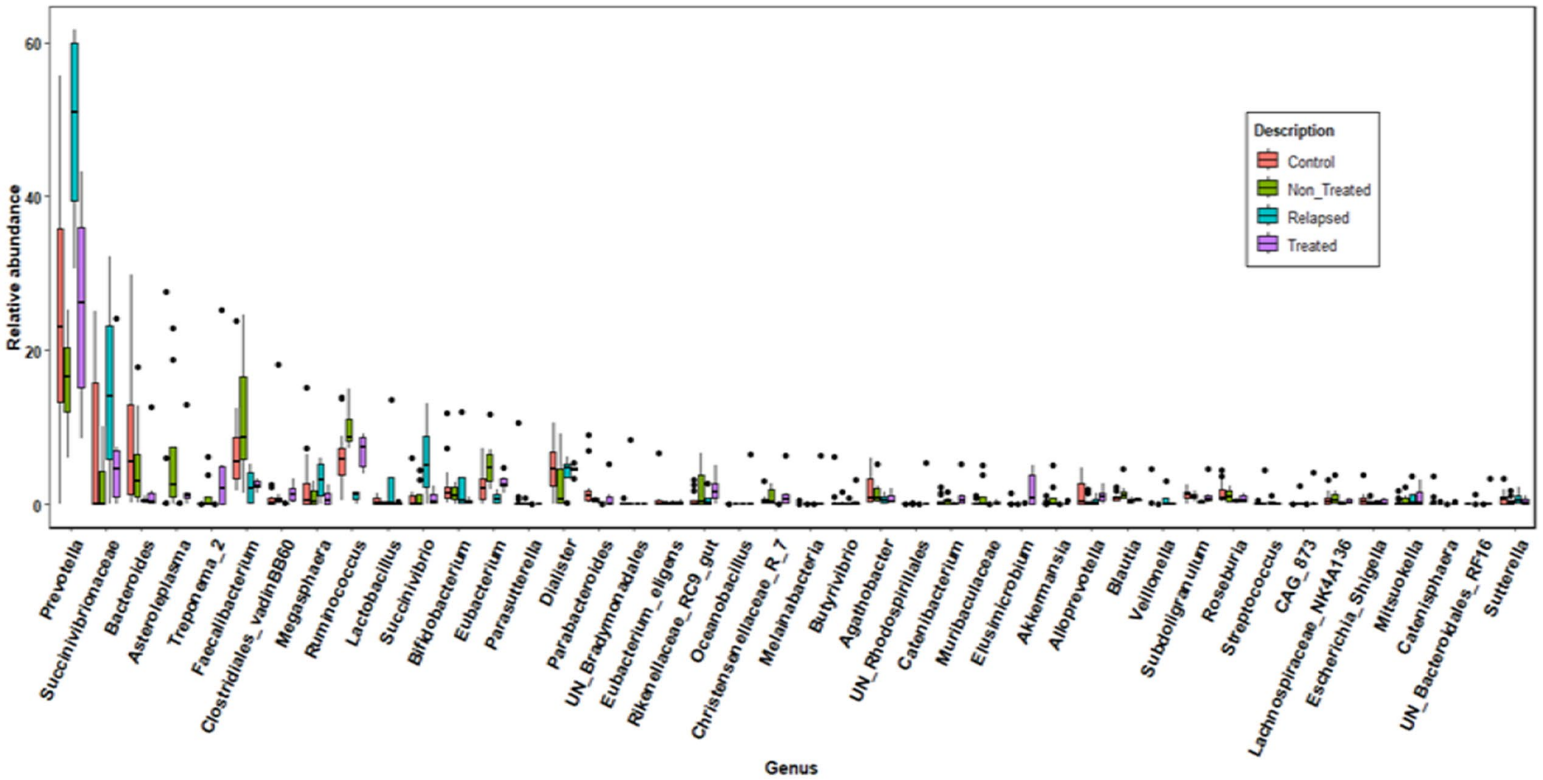
Genus-level analysis of the gut microbiome in the studied groups. Box plots illustrating the relative abundance of the dominant bacterial genera among the four study groups: Control (red), Non-Treated (green), Relapsed (blue), and Treated (purple). Each plot represents the distribution of abundance for a specific genus. The central line in each box denotes the median, while the box edges and whiskers reflect the interquartile range and variability within each group.

Genus-level analysis revealed that the *Faecalibacterium*, *Asteroeplasma*, *Eubacterium coprostanoligenes*, *Lachnospiraceae*, *Akkermansia*, and *Muribaculaceae metagenomes* were significantly predominant in the Non-Treated CHC group. *Prevotella, Bifidobacterium*, and *Lactobacillus* were more prevalent in the relapsed group, whereas *Bacteroides*, *Agathobacter,* and *Parabacteroides* were more abundant in the control group. Additionally, *Elusimicrobium*, *Christensenellaceae R-7*, *Catenibacterium*, *Oceanobacillus*, and *Candidatus Melainabacteria* were significantly more abundant in the SVR group (Figure 4).

The core genera common to all groups were *Prevotella and Faecalibacterium.* Some common core genera were shared among the different groups, but the following were exclusively found in one group: *Asteroplasma, Eubacterium coprostanoligenes, Lachnospiraceae, Akkermansia, Muribaculaceae metagenome*, and *Phascolarctabacterium*. The relapsed group had *Mitsuokella* and *Clostridium sensu stricto.* The specific core genera for the treated group that achieved SVR were *Oceanobacillus* and *Fusobacterium.* The controls included *Parasutterella*, *Collinsella*, *Escherichia Shigella*, *Lachnoclostridium* and *Coprococcus*.

### 3.4 Microbiome-clinical correlations in treatment status

**Figure 5:**
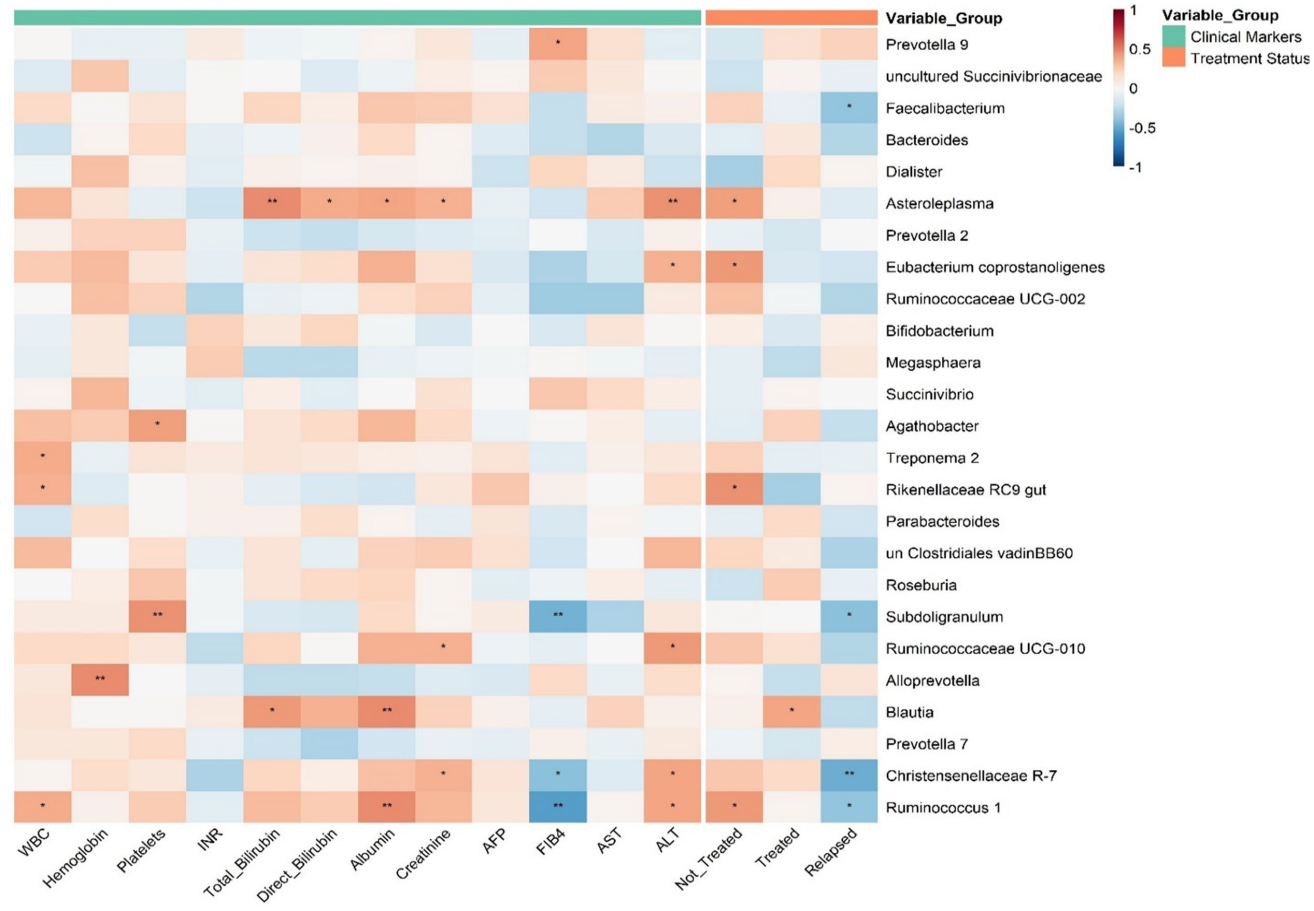
Correlations between gut microbiota and clinical parameters in HCV patients. Heatmap illustrates the Spearman correlation coefficients between the top 25 most abundant gut microbial genera and clinical and treatment-related variables. Clinical markers (e.g., liver enzymes, bilirubin, and blood counts) and treatment status groups (e.g., Treated, Relapsed, and Healthy Control) are annotated and color-coded. Asterisks indicate statistical significance (*p <* 0.05: *, *p <* 0.01: **, *p <* 0.001: ***). Positive correlations are shown in red, negative in blue, with intensity reflecting correlation strength.

Significant correlations emerged between specific microbial genera and treatment status (Figure 5). *Prevotella 9* showed positive associations with relapse status (*r* = 0.325, *p* = 0.012), while *Faecalibacterium* exhibited strong negative correlations with relapse (*r* = −0.399, *p* = 0.035). For treated patients, *Asteroeplasma* demonstrated positive correlations (*r* = 0.059, *p* = 0.002), whereas *uncultured Clostridiales vadinBB60* was positively linked to treated status (*r* = 0.090, *p* = 0.046) but negatively correlated with relapse (*r* = −0.317, *p* = 0.018). *Bacteroides* displayed inverse correlations with non-treated status (*r* = −0.118, *p* = 0.05), while *Megasphaera* showed positive associations with relapsed (*r* = 0.108, *p* = 0.0002) and healthy controls (*r* = 0.266, *p* = 0.008). Healthy controls further correlated negatively with *Asteroeplasma* (*r* = −0.405, *p* = 0.004) and positively with *Parabacteroides* (*r* = 0.070, *p* = 0.0065).

The dominant genera exhibited significant correlations with clinical biomarkers. *Prevotella and uncultured Succinivibrionaceae* showed positive correlations with hemoglobin levels. *Faecalibacterium* and *Dialister* were negatively associated with total bilirubin and AST, respectively. On the other hand, *Asteroeplasma* was positively associated with ALT. The *Eubacterium coprostanoligenes* group displayed a dual pattern: positively associated with AFP, a tumor marker, and negatively with PT/INR, a coagulation indicator. Similarly, *Ruminococcaceae UCG_002* was negatively associated with PT/INR but positively with the FIB4 fibrosis score. Lastly, *Bifidobacterium* was enriched in both treated and untreated groups, while *Megasphaera* was depleted in both.

### 3.5 Identification of biomarkers and discriminative taxa

**Figure 6:**
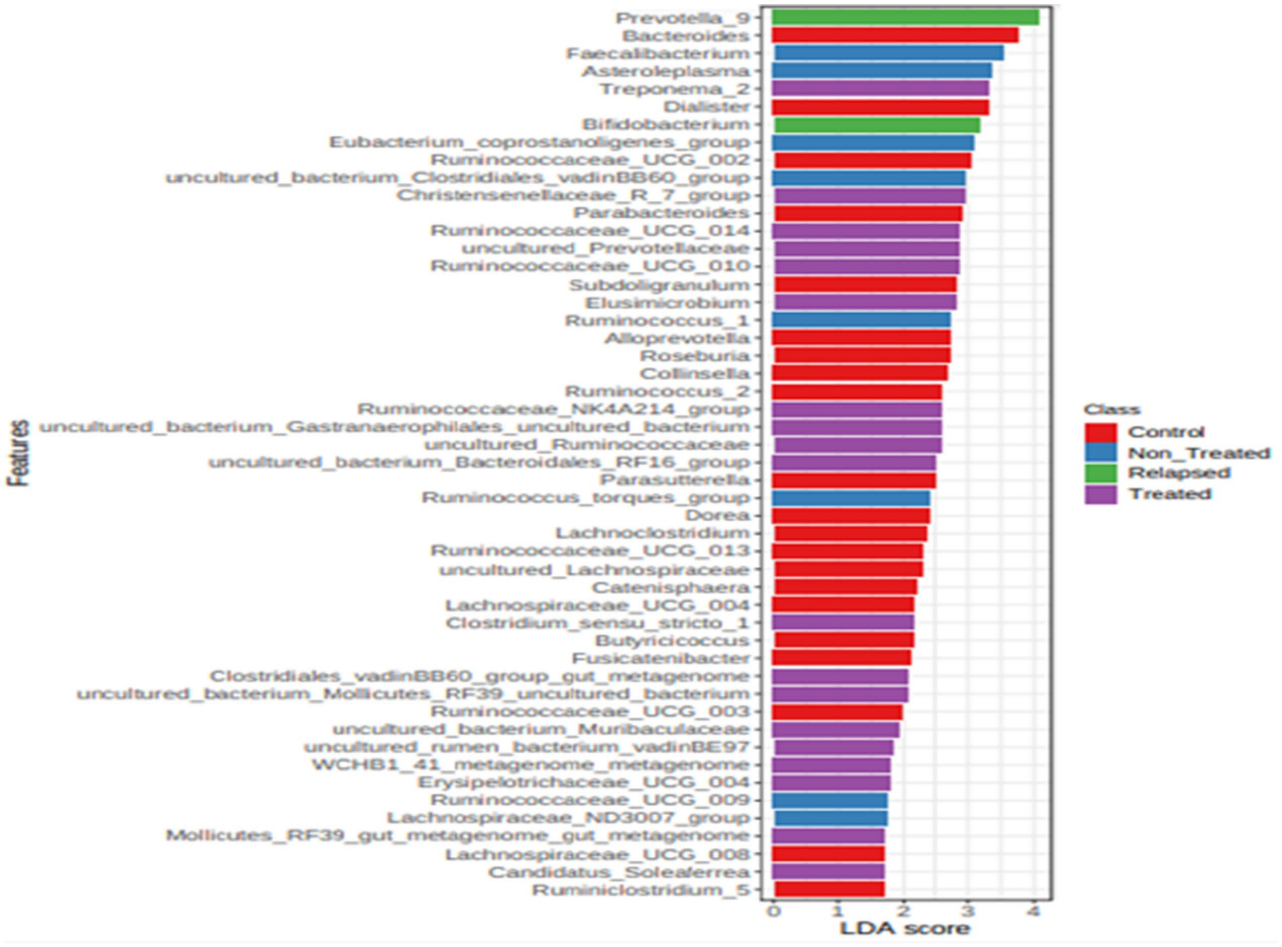
Histogram based on LEfSe analysis of discriminative taxa between the microbiomes of the four study groups. Bar chart displaying the bacterial taxa that significantly differentiate the microbiomes of the four study groups, Control (green), Non-Treated (blue), Relapsed (red), and Treated (purple), as identified by LEfSe (Linear Discriminant Analysis Effect Size) analysis. Only taxa with LDA scores ≥2.0 are shown. The *x*-axis represents the LDA score, reflecting the magnitude of each taxon’s contribution to group differentiation, while the *y*-axis lists the corresponding taxa. Color-coded bars highlight the group in which each taxon is most enriched.

Through LEfSe analysis, the significant biomarkers at the genus level for each group were determined. In the control group, *Bacteroides, Dialister,* and *Ruminococcaceae* were identified as biomarkers. In the Non-Treated CHC group, *Faecalibacterium, Asteroeplasma*, and *Eubacterium coprostanoligenes* were biomarkers. The relapsed group had *Prevotella* and *Bifidobacterium* as biomarkers, whereas the treated group had *Treponema* and *Christensenelaceae* as biomarkers (Figure 6).

## 4 Discussion

Changes in the gut microbiota play crucial roles in liver damage caused by various factors, including viruses, through the gut-liver axis (Cesaro et al., 2011). Although several studies have investigated the link between the gut microbiota composition and hepatitis C virus, the results have been inconsistent and influenced by treatment regimens (Sultan et al., 2021; Honda et al., 2021; Wellhöner et al., 2021). Research on the impact of DAAs on the gut microbiota, particularly among patients who relapse after treatment, is limited. This study aimed to evaluate the impact of CHC on the gut microbiota and to compare changes in the gut microbiota between those who achieved SVR after DAAs with those who experienced relapse.

Our findings revealed a significant increase in alpha diversity among Non-Treated CHC patients compared with other groups, which aligns with studies from Egypt and globally (Sultan et al., 2021; Hsu et al., 2022). However, several studies have shown decreased microbiota diversity in chronic HCV infections (Heidrich et al., 2018; Inoue et al., 2018). For example, Aly et al. (2016) reported lower alpha diversity in HCV patients with stage 4 disease than in eight healthy controls, whereas Ponziani et al. reported reduced alpha diversity in cirrhotic HCV patients than in healthy subjects before treatment (Ponziani et al., 2018).

In this work, the alpha diversity of patients treated with DAAs who achieved SVR significantly differed from that of patients in the nontreated CHC group and those who relapsed. The diversity of the gut microbiota was restored to a level comparable to that of the control group, with no significant difference, which is consistent with previous studies (Wellhöner et al., 2021; Hsu et al., 2022; Heidrich et al., 2018). On the other hand, DAAs did not significantly impact the gut microbiomes in other studies (Honda et al., 2021; Huang et al., 2023). The degree of underlying fibrosis plays a crucial role in gut microbiota restoration post-HCV eradication, with patients having lower fibrosis levels showing complete recovery, whereas those with higher fibrosis levels showed hindered recovery despite achieving SVR (Abd Alla et al., 2018; Pérez-Matute et al., 2019). Liver remodeling, which includes improved fibrosis and liver stiffness, occurs gradually in patients recovering from HCV, suggesting a link between liver changes and the gut microbiota (Wellhöner et al., 2021). Other studies have indicated no significant differences in gut dysbiosis between those who have cleared the virus and those who are still infected, emphasizing that HCV viremia alone does not dictate specific microbiota patterns when demographics and medical conditions, e.g., comorbidities, cirrhosis severity, and medication, are considered (Bajaj et al., 2016; Hsu et al., 2022).

In contrast, Sultan et al. (2021) reported that healthy adults had lower microbial diversity than treated HCV patients did, suggesting that treatment affects the microbiome. Similarly, Ponziani et al. (2018) reported improved alpha diversity and gut microbial composition changes a year after HCV eradication in cirrhotic patients treated with DAA regimens, which were linked to pathophysiological improvements.

Dysbiosis severity correlates with disease stage and may influence progression, but it remains unclear whether HCV infection directly alters the gut microbiota, worsening liver inflammation and fibrosis through portal endotoxemia, or if gut dysbiosis results from chronic liver inflammation and dysfunction, creating a vicious cycle rather than stemming from the viral infection itself (Sultan et al., 2021; Heidrich et al., 2018; Ponziani et al., 2018; Pérez-Matute et al., 2019).

Owing to their specificity, high effectiveness, tolerability, and short duration, concerns about long-term effects of DAA regimens on microbial communities may be alleviated (Hsu et al., 2022; Götte and Feld, 2016).

In the present study, the relapsed group presented significantly lower gut microbiota diversity than the other groups did, likely because liver dysfunction affects bile acid synthesis and secretion, which inhibits beneficial bacteria. Persistent systemic inflammation can compromise gut barrier function, allowing lipopolysaccharides (LPSs) to enter the bloodstream and trigger inflammatory responses that disrupt the gut microbiota. This imbalance may exacerbate hepatitis viral infections (Xu et al., 2015). DAAs may alter the gut flora through liver metabolism changes or immune modulation (Trifan et al., 2023; Frumento, 2025). Relapsed HCV patients are more susceptible to secondary infections due to liver dysfunction and immune impairment, and frequent antibiotic use can further diminish beneficial bacteria, reducing microbial diversity. Additionally, dietary restrictions and nutrient malabsorption may impact gut health (Virseda-Berdices et al., 2025; Petersen and Round, 2014). Although DAAs can cure HCV, they may not fully reverse T-cell exhaustion, increasing the risk of reinfection (Thimme, 2021).

HCV RNA can reappear after a SVR is achieved because the virus resides in extrahepatic sites, such as gastrointestinal mucosa cells and peripheral blood mononuclear cells (PBMCs). Even after treatment with DAAs resulting in SVR, some patients have shown occult HCV infection with a negative-strand viral genome detected in PBMCs, indicating ongoing viral replication despite successful treatment (Frumento and Ţălu, 2024; Russelli et al., 2017; Elmasry et al., 2017).

We observed distinct clustering of the gut microbiome on the basis of disease status, which contrasts with the findings of Sultan et al. (2021), who reported no significant clustering. In this study, we identified 26 bacterial phyla, with Firmicutes and Bacteroidetes being the most dominant, followed by Proteobacteria, *Spirochetes*, and *Cyanobacteria*. Firmicutes were most abundant in Non-Treated CHC patients, whereas Bacteroidetes and Proteobacteria were enriched in the relapsed group. Notably, the Firmicutes/Bacteroidetes ratio significantly differed between HCV patients and controls, with a significant decrease in the relapsed group compared with untreated CHC patients and those who achieved SVR.

In Non-Treated CHC patients, a predominance of Firmicutes was noted, echoing findings by Pérez-Matute et al. (2019), who also reported higher Actinobacteria levels in HCV-infected subjects with less fibrosis. Our study revealed a significant decrease in Actinobacteria among those who achieved SVR, accompanied by an increase in *Cyanobacteria*. Firmicutes and Proteobacteria were previously linked to inflammatory effects in the gastrointestinal tract (Tecer et al., 2020).

Aly et al. (2016) reported increased levels of Bacteroidetes in HCV patients, whereas healthy individuals presented increased Firmicutes, Proteobacteria, and Actinobacteria. Inoue et al. (2018) reported similar findings, noting increased Bacteroidetes and decreased Firmicutes in CHC patients. An analysis of 14 HCV patients revealed fluctuations in the dominance of these phyla during treatment, with Bacteroidetes and Fusobacteria decreasing while Firmicutes and *Verrucomicrobia* increased (Honda et al., 2021). In the relapsed group in this study, Bacteroidetes and Proteobacteria were the most abundant phyla. Research on the microbiota patterns of HCV-relapsed patients remains limited.

In the present study, *Bifidobacterium*, *Prevotella*, *Lactobacillus*, *Megasphaera*, and *Mitsuokella* were significantly more prevalent in the relapsed group, whereas *Faecalibacterium*, *Eubacterium coprostanoligenes*, *Asteroeplasma*, *Lachnospiraceae*, *Akkermansia*, and *Muribaculaceae* were more prevalent in the Non-Treated CHC group. Similarly, Aly et al. (2016) reported that *Faecalibacterium* and *Prevotella* were more abundant in HCV patients than in healthy individuals, whereas *Clostridium* and *Ruminococcus* were more common in healthy controls. These findings suggest that the increased Bacteroidetes in HCV patients may stem from an overabundance of *Prevotella*, which is known for its proinflammatory properties (Larsen, 2017). The heightened levels of *Prevotella* in the relapsed group could be linked to a heightened inflammatory state, exacerbating intestinal inflammation and affecting liver and systemic inflammation through signaling metabolites (Midori et al., 2024; Iljazovic et al., 2020). Additionally, impaired digestion and absorption in HCV patients may lead to increased carbohydrate levels in the intestine, promoting *Prevotella* expansion (Aly et al., 2016). Notably, Aly et al. (2016) reported a greater abundance of *Faecalibacterium prausnitzii*, which is associated with anti-inflammatory effects whereas *Akkermansia* is crucial for maintaining the intestinal mucosal barrier (Ghotaslou et al., 2023). The predominance of many of these beneficial bacteria in the nontreated CHC and relapsed groups may act as a compensatory mechanism to combat chronic inflammation and support gut health. However, the impact of DAAs on microbiota patterns and their role in relapse remain unclear and require further investigation.

Compared with healthy controls, Heidrich et al. (2018) reported that CHC patients had elevated levels of bacteria such as *Escherichia Shigella*, *Akkermansia*, *Haemophilus*, *Bifidobacterium*, *Weissella*, *Micrococcus*, *Citrobacter*, *Pediococcus*, and *Clostridium sensu stricto*. Similarly, Huang et al. (2023) noted relatively high levels of *Eubacterium*, *Ruminococcaceae*, *Alistipes*, *Agathobacter*, *Klebsiella*, and *Bifidobacterium* in the CHC group. In contrast, Ashour et al. (2022) reported that *Bifidobacterium, Ruminococcus* and some *clostridia* were more abundant in healthy controls than in HCV-infected patients. The variability among these studies may be attributed to the small sample sizes and factors influencing the gut microbiota, which are challenging to control in clinical studies, such as genetics, immune response, diet, and environmental microbial exposure (Lynch and Pedersen, 2016). In patients who achieved SVR, our findings revealed a significant increase in *Clostridium sensu stricto*, suggesting a restoration of gut homeostasis and microbial balance.

In the treatment-naive CHC group, many clusters presented significant positive correlations with gut microbiota members, including *Escherichia Shigella, Veillonella, Streptococcus, Lactobacillus,* and *Bifidobacteria*, which may contribute to disease pathogenesis. Yang et al. (2023) identified *Lactobacillus*, *Butyricimonas*, *Veillonella*, and *Escherichia-Shigella* as potential microbial markers for predicting the risk of developing viral hepatitis.

In our study, genera such as *Lactobacillus*, *Bifidobacterium*, *Treponema*, *Parasutterella*, *Veillonella*, and *Streptococcus* were positively correlated in the relapsed group, indicating disruption of intestinal integrity and increased liver inflammation. Conversely, the treated group that achieved SVR was positively correlated with beneficial bacteria responsible for intestinal integrity and health, such as *Roseburia*, *Blautia*, *Catenibacteria*, *Prevotella*, and *Parabacteroides*. Additionally, *Bacteroides*, *Megasphaera*, and *Streptococci* were negatively correlated with gut health-promoting bacteria such as *Eubacterium eligens* and *Alloprevotella*.

In the control group, several members, including the *Eubacterium eligens* group, *Akkermansia*, *Christensenellaceae R-7*, *Butyrovibrio*, and *Asteroplasma*, were positively correlated with maintaining intestinal integrity. *A. muciniphila* contributes to gut health by producing short-chain fatty acids (SCFAs), such as acetate and propionate (Lukovac et al., 2014), and enhances antimicrobial peptide synthesis and improves gut homeostasis (Ottman et al., 2017). *Eubacterium* genus members produce butyrate, which is essential for energy balance, immune regulation, colonic function, and inflammation control in the gut, whereas *Butyrivibrio*, another butyrate producer, also supports gut health (Mukherjee et al., 2020).

Correlation analyses revealed clinically relevant microbiota-host interactions. The positive association between *Prevotella* and relapse status aligns with its proinflammatory role, potentially exacerbating hepatic inflammation. Conversely, *Faecalibacterium* exhibited negative correlation with bilirubin and relapse which could be related to anti-inflammatory properties (Jiang et al., 2019; Lee et al., 2022). Intriguingly, *Eubacterium coprostanoligenes* showed dual associations, positively with AFP (a tumor marker) and negatively with PT/INR, which support its complex role in coagulation and oncogenesis. Similarly, *Ruminococcaceae UCG-002* correlated negatively with PT/INR but positively with FIB-4, implying microbial involvement in fibrosis progression (Jinato et al., 2024).

Despite its strengths, this study has limitations. The single-center design and modest sample size may limit the generalizability of our findings, particularly across diverse geographic or dietary populations. While we controlled for antibiotic/probiotic use, unmeasured confounders such as dietary habits, regional microbiome variations, and lifestyle factors could influence microbial composition. Additionally, although we correlated microbial profiles with liver function tests (e.g., ALT, AST), future studies with longitudinal designs and expanded clinical metadata (e.g., detailed dietary records, inflammatory markers) could further elucidate causal relationships between microbiota shifts and host physiology. Larger, multi-center cohorts are needed to validate these preliminary observations and explore the long-term effects of DAAs on gut-liver axis dynamics.

## 5 Conclusion

The gut microbiota was grouped on the basis of disease status, with each group exhibiting distinct microbial patterns, biomarkers, and interactions. DAAs significantly influence the diversity of the gut microbiota, resulting in beneficial restoration and reconstitution of microbial communities in the SVR group, which resemble those observed in healthy individuals.

## Data Availability

The data presented in this study are deposited in the NCBI BioProject repository, accession number PRJNA1306007. https://www.ncbi.nlm.nih.gov/bioproject/PRJNA1306007.

## References

[ref1] Abd Alla M. Gomaa A. Farrag G. A. Shikhroho M. Mousa W. Mahmoud O. (2018). Retrospective study of hepatitis c virus relapse after treatment with sofosbuvir and daclatasvir with or without ribavirin. Al-Azhar Assiut Med. J. 16:197. doi: 10.4103/AZMJ.AZMJ_22_18

[ref2] Aly A. M. Adel A. El-Gendy A. O. Essam T. M. Aziz R. K. (2016). Gut microbiome alterations in patients with stage 4 hepatitis C. Gut Pathog. 8, 1–12. doi: 10.1186/s13099-016-0124-2 27625705 PMC5020480

[ref3] Anderson M. J. (2001). A new method for non-parametric multivariate analysis of variance. Austral Ecol. 26, 32–46. doi: 10.1111/j.1442-9993.2001.01070.pp.x

[ref4] Ashour Z. Shahin R. Ali-Eldin Z. El-Shayeb M. El-Tayeb T. Bakr S. (2022). Potential impact of gut Lactobacillus acidophilus and *Bifidobacterium bifidum* on hepatic histopathological changes in non-cirrhotic hepatitis C virus patients with different viral load. Gut Pathog. 14, 25–28. doi: 10.1186/s13099-022-00501-4, PMID: 35706051 PMC9199141

[ref5] Bajaj J. S. Sterling R. K. Betrapally N. S. Nixon D. E. Fuchs M. Daita K.. (2016). HCV eradication does not impact gut dysbiosis or systemic inflammation in cirrhotic patients. Aliment. Pharmacol. Ther. 44, 638–643. doi: 10.1111/apt.13732, PMID: 27417456

[ref6] Benjamini Y. Hochberg Y. (1995). Controlling the false discovery rate: a practical and powerful approach to multiple testing. J. Roy. Stat. Soc. Ser. B (Methodol.) 57, 289–300. doi: 10.1111/j.2517-6161.1995.tb02031.x, PMID: 40771761

[ref7] Bonder A. Afdhal N. (2014). Utilization of FibroScan in clinical practice. Curr. Gastroenterol. Rep. 16:372. doi: 10.1007/s11894-014-0372-6 24452634

[ref8] Cesaro C. Tiso A. Del Prete A. Cariello R. Tuccillo C. Cotticelli G.. (2011). Gut microbiota and probiotics in chronic liver diseases. Dig. Liver Dis. 43, 431–438. doi: 10.1016/j.dld.2010.10.015, PMID: 21163715

[ref9] Dhariwal A. Chong J. Habib S. King I. L. Agellon L. B. Xia J. (2017). Microbiomeanalyst: a web-based tool for comprehensive statistical, visual and meta-analysis of microbiome data. Nucleic Acids Res. 45, W180–W188. doi: 10.1093/nar/gkx295, PMID: 28449106 PMC5570177

[ref10] Elmasry S. Wadhwa S. Bang B. R. Cook L. Chopra S. Kanel G.. (2017). Detection of occult hepatitis C virus infection in patients who achieved a sustained virologic response to direct-acting antiviral agents for recurrent infection after liver transplantation. Gastroenterology 152, 550–553.e8. doi: 10.1053/j.gastro.2016.11.002, PMID: 27838287 PMC5285320

[ref11] Elnadry M. H. Abdel-Aziz S. A. Ghareb M. Ahamad A. A. Abu Mohammed N. M. Tayel M. M. (2018). Impact of direct-acting antiviral therapy in Egyptian patients with chronic hepatitis C and liver cirrhosis. Sci. J. Al-Azhar Med. Faculty Girls 2, 181–188. doi: 10.4103/sjamf.sjamf_32_18

[ref12] Fahmy S. I. El Sherbini A. F. (1983). Determining simple parameters for social classification for health research. Bull. High Inst. Public Health. 8, 95–107.

[ref13] Fathalla Khattab A. Ammar I. Mohamed Massoud A. (2021). Assessment of liver fibrosis before and after direct acting antiviral therapy in compensated HCV related liver disease. Al-Azhar Med. J. 50, 2927–2936. doi: 10.21608/amj.2021.196431

[ref14] Frumento D. (2025). Țălu Ștefan. Interaction between human microbiota, immune system, and hepatitis C virus infection: a narrative review. Appl. Sci. 15:3157. doi: 10.3390/app15063157

[ref15] Frumento D. Ţălu Ş. (2024). Treatment with directly acting antivirals (DAAs) in HCV mono-infected and HIV-HCV co-infected patients. Microbes Infect. Dis. (In press). doi: 10.21608/mid.2024.303563.2069

[ref16] Ghotaslou R. Nabizadeh E. Memar M. Y. Law W. M. H. Ozma M. A. Abdi M.. (2023). The metabolic, protective, and immune functions of *Akkermansia muciniphila*. Microbiol. Res. 266:127245. doi: 10.1016/j.micres.2022.127245, PMID: 36347103

[ref17] Götte M. Feld J. J. (2016). Direct-acting antiviral agents for hepatitis C: structural and mechanistic insights. Nat. Rev. Gastroenterol. Hepatol. 13, 338–351. doi: 10.1038/nrgastro.2016.60, PMID: 27147491

[ref18] Grüner N. Mattner J. (2021). Bile acids and microbiota: multifaceted and versatile regulators of the liver–gut axis. Int. J. Mol. Sci. 22, 1–17. doi: 10.3390/ijms22031397, PMID: 33573273 PMC7866539

[ref19] Guilliams M. Bonnardel J. Haest B. Vanderborght B. Wagner C. Remmerie A.. (2022). Spatial proteogenomics reveals distinct and evolutionarily conserved hepatic macrophage niches. Cell 185, 379–396.e38. doi: 10.1016/j.cell.2021.12.018, PMID: 35021063 PMC8809252

[ref20] Heidrich B. Vital M. Plumeier I. Döscher N. Kahl S. Kirschner J.. (2018). Intestinal microbiota in patients with chronic hepatitis C with and without cirrhosis compared with healthy controls. Liver Int. 38, 50–58. doi: 10.1111/liv.13485, PMID: 28561276

[ref21] Honda T. Ishigami M. Yamamoto K. Takeyama T. Ito T. Ishizu Y.. (2021). Changes in the gut microbiota after hepatitis C virus eradication. Sci. Rep. 11:23568. doi: 10.1038/s41598-021-03009-0, PMID: 34876650 PMC8651745

[ref22] Hsu Y. C. Chen C. C. Lee W. H. Chang C. Y. Lee F. J. Tseng C. H.. (2022). Compositions of gut microbiota before and shortly after hepatitis C viral eradication by direct antiviral agents. Sci. Rep. 12, 5481–5410. doi: 10.1038/s41598-022-09534-w, PMID: 35361930 PMC8971444

[ref23] Huang P. Y. Chen C. H. Tsai M. J. Yao C. C. Wang H. M. Kuo Y. H.. (2023). Effects of direct anti-viral agents on the gut microbiota in patients with chronic hepatitis C. J. Formos. Med. Assoc. 122, 157–163. doi: 10.1016/j.jfma.2022.08.022, PMID: 36155707

[ref24] Iljazovic A. Roy U. Gálvez E. J. C. Lesker T. R. Zhao B. Gronow A.. (2020). Perturbation of the gut microbiome by *Prevotella* spp. enhances host susceptibility to mucosal inflammation. Mucosal Immunol. September 2019 14, 113–124. doi: 10.1038/s41385-020-0296-4 32433514 PMC7790746

[ref25] Illumina (2013). 16S metagenomic sequencing library. Illumina.com. 1–28. Available online at: http://support.illumina.com/content/dam/illumina-support/documents/documentation/chemistry_documentation/16s/16s-metagenomic-library-prep-guide-15044223-b.pdf

[ref26] Inoue T. Nakayama J. Moriya K. Kawaratani H. Momoda R. Ito K.. (2018). Gut dysbiosis associated with hepatitis C virus infection. Clin. Infect. Dis. 67, 869–877. doi: 10.1093/cid/ciy205, PMID: 29718124

[ref27] Jiang J. W. Chen X. H. Ren Z. Zheng S. S. (2019). Gut microbial dysbiosis associates hepatocellular carcinoma via the gut-liver axis. Hepatobiliary Pancreat. Dis. Int. 18, 19–27. doi: 10.1016/j.hbpd.2018.11.002, PMID: 30527903

[ref28] Jinato T. Anuntakarun S. Satthawiwat N. Chuaypen N. Tangkijvanich P. (2024). Distinct alterations of gut microbiota between viral- and non-viral-related hepatocellular carcinoma. Appl. Microbiol. Biotechnol. 108, 1–14. doi: 10.1007/s00253-023-12845-1 38183473 PMC10771587

[ref29] Kakiyama G. Pandak W. M. Gillevet P. M. Hylemon P. B. Heuman D. M. Daita K.. (2013). Modulation of the fecal bile acid profile by gut microbiota in cirrhosis. J. Hepatol. 58, 949–955. doi: 10.1016/j.jhep.2013.01.003, PMID: 23333527 PMC3936319

[ref30] Ladenheim J. Garcia G. Titzer D. Herzenberg H. Lavori P. Edson R.. (1995). Effect of sulindac on sporadic colonic polyps. Gastroenterology 108, 1083–1087. doi: 10.1016/0016-5085(95)90206-6, PMID: 7698575

[ref31] Larsen J. M. (2017). The immune response to Prevotella bacteria in chronic inflammatory disease. Immunology 151, 363–374. doi: 10.1111/imm.12760, PMID: 28542929 PMC5506432

[ref32] Lee P. C. Wu C. J. Hung Y. W. Lee C. J. Chi C. T. Lee I. C.. (2022). Gut microbiota and metabolites associate with outcomes of immune checkpoint inhibitor-treated unresectable hepatocellular carcinoma. J. Immunother. Cancer 10:e004779. doi: 10.1136/jitc-2022-004779, PMID: 35738801 PMC9226985

[ref33] Lukovac S. Belzer C. Pellis L. Keijser B. J. de Vos W. M. Montijn R. C.. (2014). Differential modulation by *Akkermansia muciniphila* and *Faecalibacterium prausnitzii* of host peripheral lipid metabolism and histone acetylation in mouse gut organoids. mBio 5:e01438–14. doi: 10.1128/mBio.01438-14, PMID: 25118238 PMC4145684

[ref34] Lynch S. V. Pedersen O. (2016). The human intestinal microbiome in health and disease. N. Engl. J. Med. 375, 2369–2379. doi: 10.1056/NEJMra1600266, PMID: 27974040

[ref35] Martinello M. Grebely J. Petoumenos K. Gane E. Hellard M. Shaw D.. (2017). Hcv reinfection incidence among individuals treated for recent infection. J. Viral Hepat. 24:359. doi: 10.1111/jvh.12666, PMID: 28027424 PMC5400730

[ref36] Martinello M. Naggie S. Rockstroh J. K. Matthews G. V. (2023). Direct-acting antiviral therapy for treatment of acute and recent hepatitis C virus infection: a narrative review. Clin. Infect. Dis. 77, S238–S244. doi: 10.1093/cid/ciad344, PMID: 37579203

[ref37] Midori Y. Nosaka T. Hiramatsu K. Akazawa Y. Tanaka T. Takahashi K.. (2024). Isolation of mucosa-associated microbiota dysbiosis in the ascending colon in hepatitis C virus post-sustained virologic response cirrhotic patients. Front. Cell. Infect. Microbiol. 14, 1–13. doi: 10.3389/fcimb.2024.1371429, PMID: 38650735 PMC11033736

[ref38] Mouzaki M. Wang A. Y. Bandsma R. Comelli E. M. Arendt B. M. Zhang L.. (2016). Bile acids and dysbiosis in non-alcoholic fatty liver disease. PLoS One 11, 1–13. doi: 10.1371/journal.pone.0151829 PMC4874546 27203081

[ref39] Mukherjee A. Lordan C. Ross R. P. Cotter P. D. (2020). Gut microbes from the phylogenetically diverse genus *Eubacterium* and their various contributions to gut health. Gut Microbes 12:1802866. doi: 10.1080/19490976.2020.1802866, PMID: 32835590 PMC7524325

[ref40] Nawaz A. Manzoor A. Ahmed S. Ahmed N. Abbas W. Mir M. A.. (2023). Therapeutic approaches for chronic hepatitis C: a concise review. Front. Pharmacol. 14, 1–8. doi: 10.3389/fphar.2023.1334160 PMC10811011 38283838

[ref41] Ottman N. Reunanen J. Meijerink M. Pietila T. E. Kainulainen V. Klievink J.. (2017). Pili-like proteins of *Akkermansia muciniphila* modulate host immune responses and gut barrier function. PLoS One 12:e0173004. doi: 10.1371/journal.pone.0173004, PMID: 28249045 PMC5332112

[ref42] Pérez-Matute P. Íñiguez M. Villanueva-Millán M. J. Recio-Fernández E. Vázquez A. M. Sánchez S. C.. (2019). Short-term effects of direct-acting antiviral agents on inflammation and gut microbiota in hepatitis C-infected patients. Eur. J. Intern. Med. 67, 47–58. doi: 10.1016/j.ejim.2019.06.005, PMID: 31221551

[ref43] Petersen C. Round J. L. (2014). Defining dysbiosis and its influence on host immunity and disease. Cell Microbiol. 16, 1024–1033. doi: 10.1111/cmi.12308, PMID: 24798552 PMC4143175

[ref44] Ponziani F. R. Putignani L. Paroni Sterbini F. Petito V. Picca A. Del Chierico F.. (2018). Influence of hepatitis C virus eradication with direct-acting antivirals on the gut microbiota in patients with cirrhosis. Aliment. Pharmacol. Ther. 48, 1301–1311. doi: 10.1111/apt.15004, PMID: 30345704

[ref45] Qin N. Yang F. Li A. Prifti E. Chen Y. Shao L.. (2014). Alterations of the human gut microbiome in liver cirrhosis. Nature 513, 59–64. doi: 10.1038/nature13568, PMID: 25079328

[ref46] Russelli G. Pizzillo P. Iannolo G. Barbera F. Tuzzolino F. Liotta R.. (2017). HCV replication in gastrointestinal mucosa: potential extra-hepatic viral reservoir and possible role in HCV infection recurrence after liver transplantation. PLoS One 12, 1–30. doi: 10.1371/journal.pone.0181683, PMID: 28750044 PMC5531480

[ref47] Sultan S. El-Mowafy M. Elgaml A. El-Mesery M. El Shabrawi A. Elegezy M.. (2021). Alterations of the treatment-naive gut microbiome in newly diagnosed hepatitis C virus infection. ACS Infect Dis. 7, 1059–1068. doi: 10.1021/acsinfecdis.0c00432, PMID: 33119247

[ref48] Tecer D. Gogus F. Kalkanci A. Erdogan M. Hasanreisoglu M. Ergin Ç.. (2020). *Succinivibrionaceae* is dominant family in fecal microbiota of Behçet’s syndrome patients with uveitis. PLoS One 15:e0241691. doi: 10.1371/journal.pone.0241691, PMID: 33125440 PMC7598488

[ref49] Thimme R. (2021). T cell immunity to hepatitis C virus: lessons for a prophylactic vaccine. J. Hepatol. 74, 220–229. doi: 10.1016/j.jhep.2020.09.022, PMID: 33002569

[ref50] Trifan A. Cuciureanu T. Nastasa R. Stratina E. Zenovia S. Muzica C. M.. (2023). Changes in components of metabolic syndrome after antiviral eradication in hepatitis C virus infection. Life. 13, 1–11. doi: 10.3390/life13020534, PMID: 36836890 PMC9959799

[ref51] Virseda-Berdices A. Martín-Escolano R. Berenguer J. González-García J. Brochado-Kith O. Rojo D.. (2025). Metabolomic changes associated with the change in HVPG after DAAs therapy in HCV cirrhotic patients. Liver Int. 45:e16204. doi: 10.1111/liv.16204 39708286

[ref52] Waked I. Esmat G. Elsharkawy A. El-Serafy M. Abdel-Razek W. Ghalab R.. (2020). Screening and treatment program to eliminate hepatitis C in Egypt. N. Engl. J. Med. 382, 1166–1174. doi: 10.1056/NEJMsr1912628, PMID: 32187475

[ref53] Wellhöner F. Döscher N. Woelfl F. Vital M. Plumeier I. Kahl S.. (2021). Eradication of chronic HCV infection: improvement of Dysbiosis only in patients without liver cirrhosis. Hepatology 74, 72–82. doi: 10.1002/hep.31700, PMID: 33411981

[ref54] WHO Global Hepatitis Report (2022). Available online at: https://www.who.int/news-room/fact-sheets/detail/hepatitis-c (Accessed January 14, 2024).

[ref55] Wong J. M. de Souza R. Kendall C. W. Emam A. J. D. J. (2006). No title colonic health: fermentation and short chain fatty acids. J. Clin. Gastroenterol. 40, 235–243. doi: 10.1097/00004836-200603000-00015, PMID: 16633129

[ref56] Xu D. Huang Y. Wang J. (2015). Gut microbiota modulate the immune effect against hepatitis B virus infection. Eur. J. Clin. Microbiol. Infect Dis. 34, 2139–2147. doi: 10.1007/s10096-015-2464-0, PMID: 26272175

[ref57] Yang X. Mai H. Zhou J. Li Z. Wang Q. Lan L.. (2023). Alterations of the gut microbiota associated with the occurrence and progression of viral hepatitis. Front. Cell. Infect. Microbiol. 13:1119875. doi: 10.3389/fcimb.2023.1119875, PMID: 37342245 PMC10277638

[ref58] Yoshida E. M. Sulkowski M. S. Gane E. J. Herring R. W. Ratziu V. Ding X.. (2015). Concordance of sustained virological response 4, 12, and 24 weeks post-treatment with sofosbuvir-containing regimens for hepatitis C virus. Hepatology 61, 41–45. doi: 10.1002/hep.27366, PMID: 25314116

[ref59] Zheng R. Wang G. Pang Z. Ran N. Gu Y. Guan X.. (2020). Liver cirrhosis contributes to the disorder of gut microbiota in patients with hepatocellular carcinoma. Cancer Med. 9, 4232–4250. doi: 10.1002/cam4.3045, PMID: 32281295 PMC7300425

